# Fermented Bamboo Fiber Improves Productive Performance by Regulating Gut Microbiota and Inhibiting Chronic Inflammation of Sows and Piglets during Late Gestation and Lactation

**DOI:** 10.1128/spectrum.04084-22

**Published:** 2023-04-12

**Authors:** Chuansong Sun, Rui Song, Jianyong Zhou, Yubiao Jia, Jianjun Lu

**Affiliations:** a Institute of Feed Science, College of Animal Sciences, Zhejiang University, Hangzhou, China; b The National Engineering Laboratory for Feed Safety and Pollution Prevention and Controlling, National Development and Reform Commission, Zhejiang University, Hangzhou, China; c Key Laboratory of Molecular Animal Nutrition, Ministry of Education, Zhejiang University, Hangzhou, China; d Key Laboratory of Animal Nutrition and Feed Science, Ministry of Agriculture and Rural Affairs, Zhejiang University, Hangzhou, China; e Key Laboratory of Animal Feed and Nutrition of Zhejiang Province, Zhejiang University, Hangzhou, China; Nanjing Agricultural University

**Keywords:** fermented bamboo fiber, sows, piglets, gut microbiota, inflammation, immunity

## Abstract

Sows exhibit metabolic syndrome and significant changes in intestinal microbiota during late gestation and lactation, affecting sow performance and piglet health. Dietary fiber (DF) is widely applied to improve sow performance by modulating gut microbiota and their by-products. Here, 60 sows were randomly allocated to groups, including CON (8% wheat bran), FBF-1 (1% fermented bamboo fiber), FBF-2 (2.5% fermented bamboo fiber), and FBF-3 (4% fermented bamboo fiber) from day 80 of gestation (G80d) to the end of lactation (L21d). Compared with CON, the FBF-3 diet decreased lactation backfat loss, increased average daily feed intake (ADFI) during lactation, and the weight gain of piglets, while supplementation of FBF increased fecal water content and reduced the rate of constipation in sows. Further, the yield and quality of milk of sows in FBF groups were improved. The FBF-3 diet significantly reduced markers of intestinal permeability (diamine oxidase and endotoxin) and systemic inflammation (interleukin-6 [IL-6] and tumor necrosis factor alpha) in sow serum during lactation, while it increased the anti-inflammatory marker (IL-10). Similarly, the piglets in the FBF-2 and FBF-3 groups had lower levels of IL-6 and higher levels of IgG, IgM, and insulin-like growth factor in serum. In addition, sows fed the 4% FBF diet had higher levels of acetate, propionate, butyrate, and total short-chain fatty acids (SCFAs) in feces than CON, and total SCFAs were promoted in piglets from the FBF-3 group. Spearman correlation analysis showed that immunity, inflammation, and intestinal microbiota are closely related to sow performance, which can affect piglet growth. The potential mechanism could be that FBF promoted the enrichment of beneficial genera such as *Lachnospira*, *Lachnospiracea_XPB1014_Group*, and *Roseburia* and the production of SCFAs in the sow’s intestine, and reduced the relative abundance of harmful bacteria such as *Fusobacterium*, *Sutterellaceae*, and *Sutterella*. Meanwhile, the intake of FBF by sows affected the gut microbial composition of their offspring piglets, significantly increasing the relative abundance of beneficial bacteria *Alistipes* and *Lachnoclostridium* and decreasing the relative abundance of pathogenic bacteria *Trueperella* among colonic microorganisms.

**IMPORTANCE** Dietary fiber is widely applied in the nutrition of sows due to its potential value in improving performance and intestinal health. Fermented bamboo fiber, rich in dietary fiber, has not been fully evaluated to be used in sow diets. Sows mobilize body reserves during gestation and lactation due to nutrients being prioritized for lactation purposes while feeding piglets, which generally leads to metabolism and immunity undergoing drastic changes. The main manifestations are increased inflammation and intestinal permeability and disturbed intestinal flora, which ultimately reduces the ADFI and milk quality, thus affecting the growth of piglets. The study described here is the first attempt to provide FBF for sows in late gestation and lactation can reverse this process. The 4% FBF was initially explored to have the most significantly beneficial effect. It provides a potentially effective method for dietary modification to control the gut microbiota and its metabolites to improve sow and piglet health. Moreover, the sow-piglet model offers a reference for investigating the impact of dietary fiber on the intestinal health of human mothers and infants.

## INTRODUCTION

Sows frequently face metabolic syndrome during late gestation and lactation because of substantial immunological and metabolic changes (1, 2). The extreme catabolism and anabolism caused by milk production during lactation might further contribute to these symptoms in sows (3). Prolonged exposure to inflammation can lead to diseases such as constipation, abortion, preterm labor, and perinatal asphyxia (4). Sows undergo complex metabolism and immune system changes, resulting in insufficient average daily feed intake (ADFI) during lactation as one of the core problems limiting reproductive performance and directly affecting the health of piglets (5, 6). Therefore, reducing inflammatory responses and metabolic disturbances during late gestation and lactation to ensure normal metabolic and immune changes is essential for the performance of the sows and piglets.

The microbiota of pregnant and lactating sows also undergoes significant changes consistent with metabolic and immune changes. These microbiota changes may also be involved in the metabolic processes of pregnant animals, leading to the development of pregnancy complications and affecting offspring health (7). In general, whether intestinal microbiota is beneficial to host health is mostly determined by its metabolites, such as short-chain fatty acids (SCFAs), derived from the fermentation of dietary fiber in diets, which can enhance the production of mucus and antimicrobial peptides and increase the expression of tight-junction proteins (8, 9). Dietary fiber as a heterogeneous class of carbohydrates is resistant to digestion by mammalian enzymes in the small intestine but largely fermented by microbiota in the hindgut (10). Dietary fiber (DF) deprivation is associated with gut dysbiosis, leading to gut inflammation, colon cancer, obesity, and type 2 diabetes (7, 11). Therefore, supplementing with DF has been dramatically investigated to benefit balanced intestinal microbiota, stay gut health, and modulate immunity (3). A high intake of DF during pregnancy may increase food intake during lactation (12). In addition to the direct effect of DF on the sow intestinal flora, a new strategy adds fiber to the mother’s diet in an attempt to improve the microbiota of the offspring (13). The microbiota seems to exert effects on the next generation, via maternal microbiota and immune responses (14). Evidence suggests that the establishment of the fetus microflora could already start *in utero*, transferred from maternal intestinal microbiota (15, 16). Maternal microbes are transferred when the infant comes in contact with the mother’s feces and vaginal microbiota and initiates the transfer of the infant’s colonization process, which is essential for establishing the microbiota of the offspring (17, 18). For instance, babies born through the vagina acquire the mother’s vaginal microbial flora, including Escherichia coli, *Lactobacillus*, *Bifidobacterium*, and *Bacteroides* (19). DF as a potent dietary modulator of gut microbiota in mothers is strongly associated with gut microbiota composition during pregnancy and lactation (20).

China is the largest bamboo-producing country and has about 300 species in 44 genera, occupying 3% of the global forest area, and the area of Moso bamboo (*Phyllostachys edulis*) accounts for about 74% of the total bamboo forest area (21, 22). To improve the comprehensive utilization of bamboo resources, the health-promoting effects of bamboo have been extensively studied. All parts of the bamboo plant, such as rhizome, stems, shavings, leaves, roots, shoots, and seeds, exhibit clinical applications. Four arabinoxylan oligosaccharides have been identified in bamboo culms, and arabinoxylan oligosaccharides in certain bamboo leaves help maintain intestinal flora homeostasis (23). Bamboo stem extracts like *p*-coumaric acid, myricetin, and flavonoids have helpful effects on antibacterial, antiobesity, anti-inflammation, cardiovascular, metabolic, and neurological disorders (23, 24). Bamboo shoot dietary fiber has several biological functions, such as antioxidant activity, anti-inflammatory effect, antidiabetic activity, and obesity prevention (25). Bamboo culm has rich DF mainly consisting of cellulose, hemicellulose, lignin, and polysaccharides, accounting for approximately 79 to 89% on weight basis (26–28). O-acetylated xylan obtained from bamboo altered the cecum microbiota of mice and effectively relieved constipation (29).

Although the health-promoting effects of bamboo shoots and leaves have been extensively studied, the beneficial effects of dietary fiber from Moso bamboo culms have not been well studied. In the present study, fermented bamboo fiber (FBF) was supplemented in the diets of sows during late gestation and lactation to study the effects of different levels of FBF on gut microbiota, inflammation, intestinal permeability, and immunity in sows and piglets. We hypothesized that supplementation with FBF in a sow diet could improve the performance of both the sow and its piglets by alleviating intestinal and systemic inflammation, improving immune performance and regulating the gut microbiota.

## RESULTS

### Effects of supplementation with fermented bamboo fiber during late gestation and lactation on the performance of sows and piglets.

**FIG 1:**
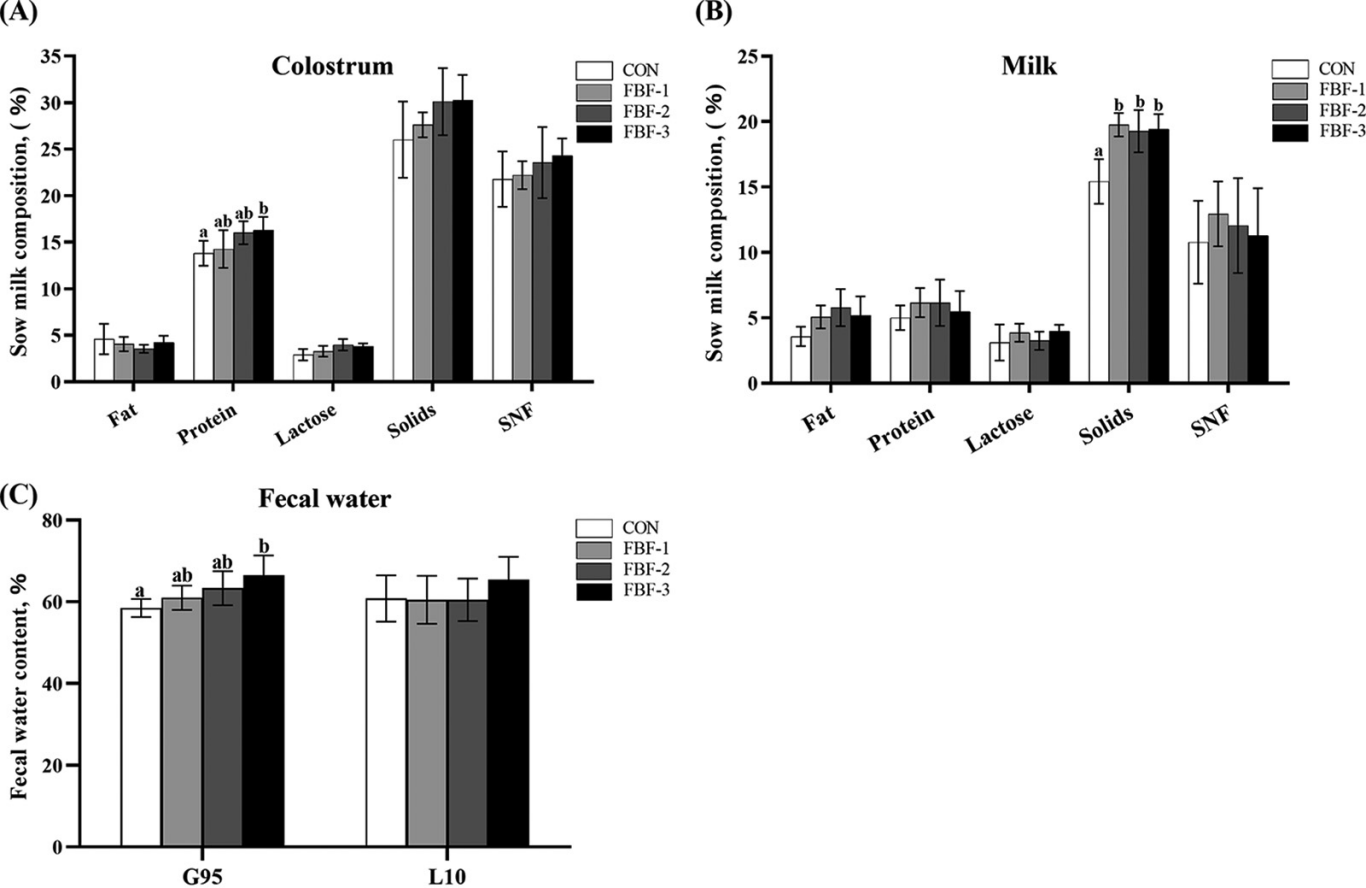
Effects of fermented bamboo fiber on the nutritional composition of sow milk and sow fecal water content. (A) Nutritional composition of colostrum on day 1 of lactation. (B) Nutritional composition of milk on day 7 of lactation. (C) Fecal water content on day 95 of gestation and day 10 of lactation. The data are presented as means ± the standard errors of the mean (SEM; *n* = 6). Different letters mean significant differences (*P < *0.05). CON, wheat bran diet; FBF-1, 1% fermented bamboo fiber diet; FBF-2, 2.5% fermented bamboo fiber diet; FBF-3, 4% fermented bamboo fiber diet; SNF, solids (nonfat).

As shown in Table 1, assessment of sow backfat thickness during the reproductive cycle, no significant differences on gestation day 85(G85), lactation day 1 (L1d), and lactation day 21 (L21d). There is an upward trend in ADFI (*P < *0.01) and a significant decrease in the backfat loss (*P < *0.05) was observed in the FBF-2 and FBF-3 in contrast with the CON. Besides, sows in the FBF groups showed a lower rate of constipation (*P < *0.05). As shown in Fig. 1C that on G95d, supplementation with 4% FBF increased the fecal water content of the sows (*P < *0.05). However, evaluation of sow reproductive performance demonstrated no significant differences in total litter size (*P = *0.86), live litter size (*P = *0.88), newborn body weight (*P = *0.71), or newborn litter weight (*P = *0.64) among the treatments. For piglets, 4% FBF significantly increased the piglet weaning weight and litter weight gain (*P < *0.05) and decreased piglet diarrhea incidence (*P < *0.05). Compared to CON, adding 4% FBF showed remarkable improvement in the protein of colostrum (*P < *0.01, Fig. 1A) and dietary FBF treatments remarkably increased the solid of ordinary milk (*P < *0.05, Fig. 1B). These results indicated that supplementing FBF during late pregnancy and lactation can improve ADFI and reproductive performance and decrease the constipation rate.

**TABLE 1:** Effects of inclusion of various proportions of fermented bamboo fiber on the performances of the lactating sows and piglets^*a*^

Animal and characteristics	Diet				SEM	*P*
	CON	FBF-1	FBF-2	FBF-3		
Sows^*b*^						
G85d backfat thickness, mm	17.78	18.52	18.19	18.04	0.22	0.37
L1d backfat thickness, mm	17.68	18.39	17.52	17.71	0.21	0.63
Weaning (L21d) backfat thickness, mm	16.49	16.53	17.00	17.31	0.27	0.35
Lactation backfat loss	1.83^A^	0.84^AB^	0.67^AB^	0.50^B^	0.21	0.05
ADFI, kg/days	6.37^A^	6.87^AB^	7.22^BC^	7.56d	0.14	0.01
Constipation rate, %	6.25^A^	5.61^B^	5.52^B^	5.15^B^	0.12	0.004
Piglets^*c*^						
No. at birth, total	12.60	13.20	12.00	12.20	0.49	0.86
No. at birth, live	11.80	12.00	11.20	11.20	0.41	0.88
No. of stillbirths	0.88	1.20	0.80	1.00	0.21	0.91
No. at weaning	9.25	9.50	9.25	9.75	0.38	0.97
Wt at birth, kg	1.22	1.30	1.20	1.26	0.30	0.71
Wt at weaning, kg	5.42^A^	5.57^AB^	5.78^AB^	6.13^C^	0.10	0.05
Litter wt at birth, kg	14.4	15.45	13.42	14.16	0.54	0.64
Litter wt at weaning, kg	53.35^A^	55.70^AB^	57.40^AB^	60.60^C^	1.21	0.04
Diarrhea incidence, %	1.32^A^	1.12^AB^	1.07^BC^	1.02^C^	0.11	0.01

a Mean values within a row with different superscript letters differ at *P *< 0.05. CON, wheat bran diet; FBF-1, 1% fermented bamboo fiber diet; FBF-2, 2.5% fermented bamboo fiber diet; FBF-3, 4% fermented bamboo fiber diet.

b Backfat loss = parturition backfat – weaning backfat; ADFI of the sows were recorded from parturition until weaning (21 days).

c Piglet weight at birth = litter weight at birth/litter size at birth (live); piglet weight at weaning = litter weight at weaning/litter size at weaning (live); diarrhea incidence = total diarrhea piglets/litter size at birth (live) × trial days.

### Effects of FBF diets on intestinal permeability, inflammatory response, and immune performance in sows and piglets.

**FIG 2:**
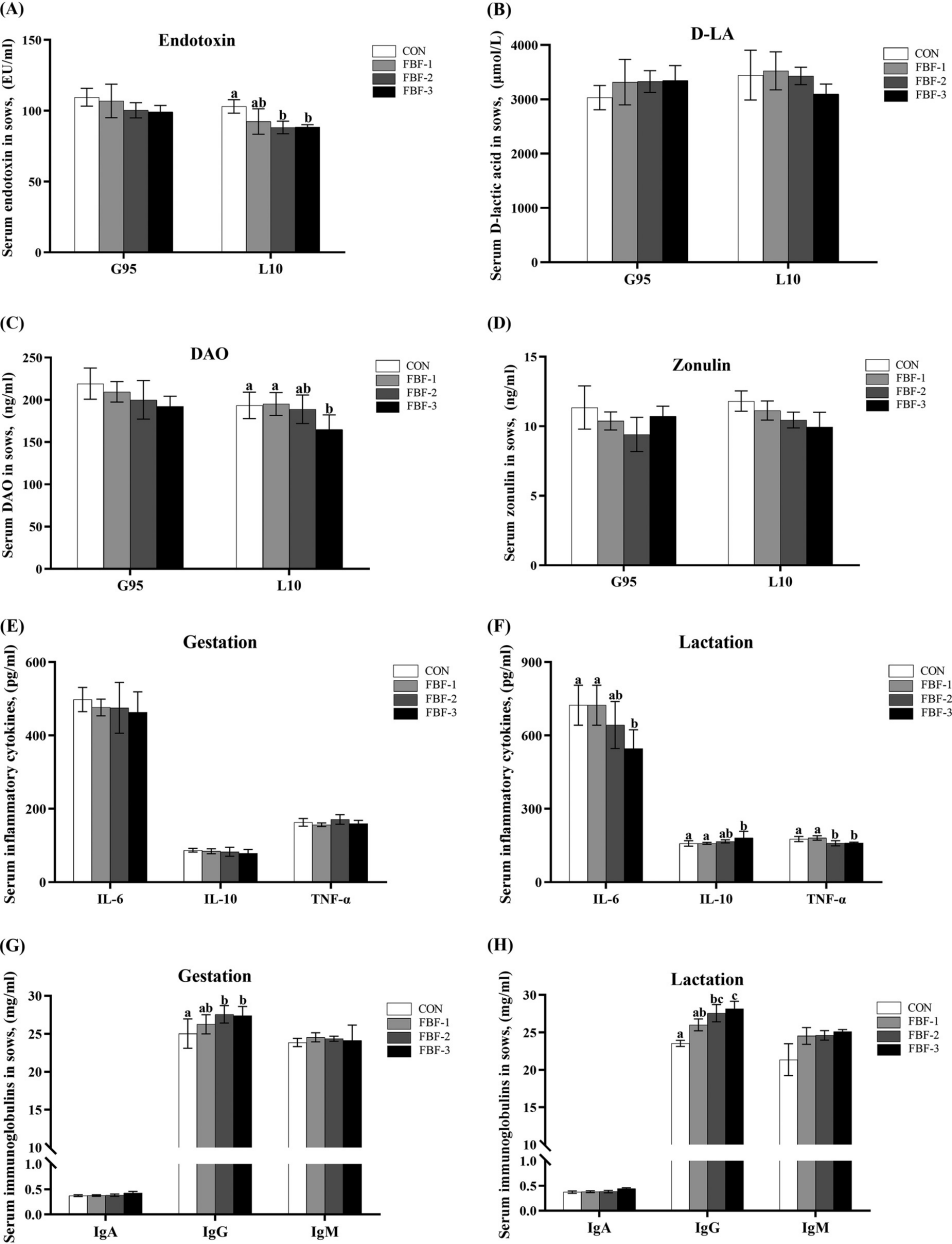
Effects of fermented bamboo fiber diet on sow serum indicators. (A) Serum endotoxin. (B) Serum D-LA(D-Lactate). (C) Serum DAO (diamine oxidase). (D) Serum zonulin. (E) Serum inflammatory cytokines (IL-6, IL-10, and TNF-α) on day 95 of gestation. (F) Serum inflammatory cytokines (IL-6, IL-10, and TNF-α) on day 10 of lactation. (G) Serum immunoglobulins (IgA, IgG, and IgM) on day 95 of gestation. (H) Serum immunoglobulins (IgA, IgG, and IgM) on day 10 of lactation. The data are presented as means ± the SEM (*n* = 6). Different letters mean significant differences (*P < *0.05). CON wheat bran diet; FBF-1, 1% fermented bamboo fiber diet; FBF-2, 2.5% fermented bamboo fiber diet; FBF-3, 4% fermented bamboo fiber diet.

**FIG 3:**
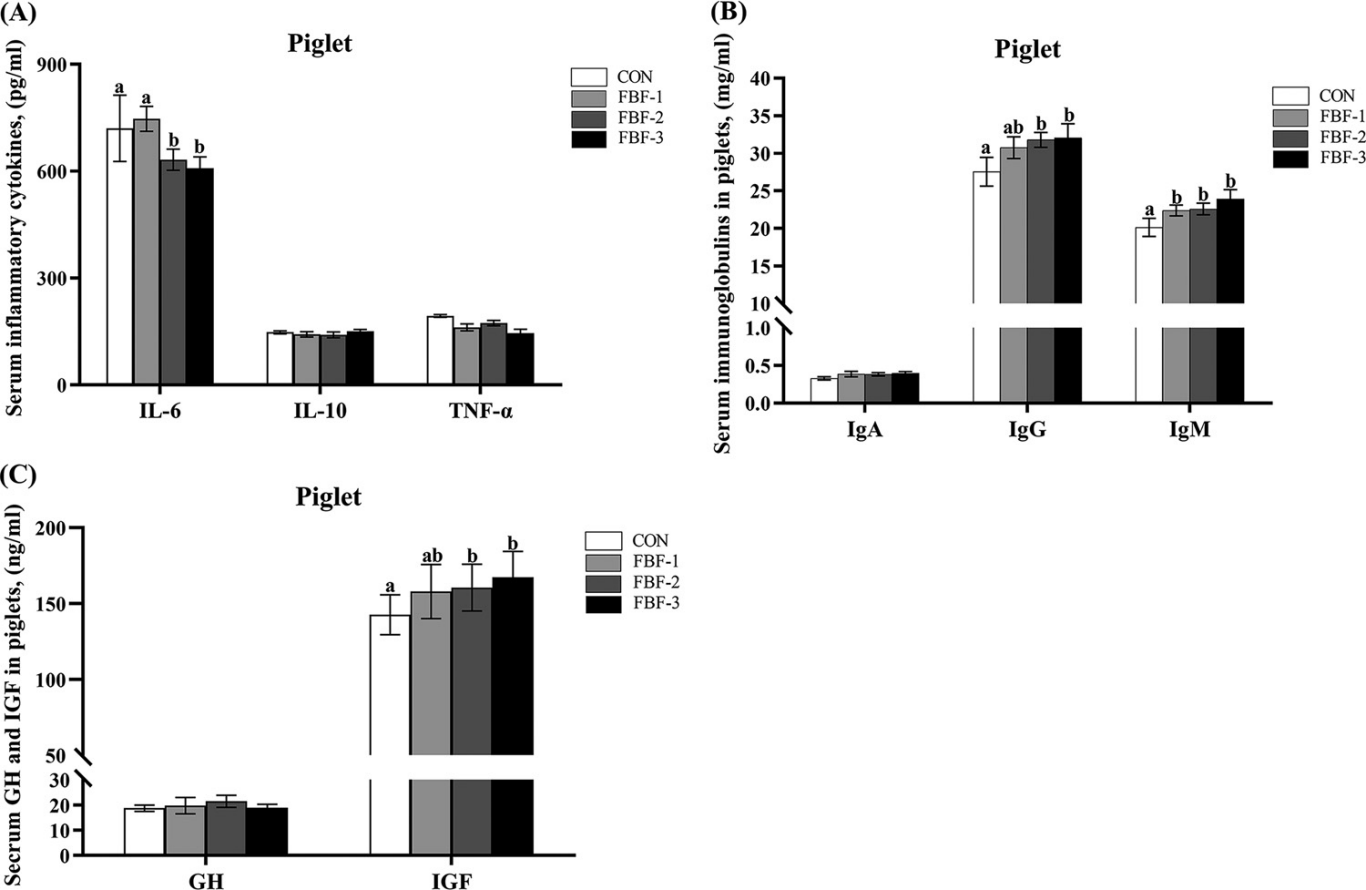
Effects of fermented bamboo fiber diet on piglet serum indicators. (A) Serum inflammatory cytokines (IL-6, IL-10, and TNF-α). (B) Serum immunoglobulins (IgA, IgG, and IgM). (C) Serum growth hormone (GH) and insulin-like growth factor (IGF). The data are presented as means ± the SEM (*n* = 6). Different letters mean significant differences (*P < *0.05). CON wheat bran diet; FBF-1, 1% fermented bamboo fiber diet; FBF-2, 2.5% fermented bamboo fiber diet; FBF-3, 4% fermented bamboo fiber diet.

Given our findings that FBF supplementation could increase the fecal water content and reduce the sows constipation rate. Accordingly, we next conducted a systematic evaluation of four biomarkers related to gut permeability (D-lactic acid [D-LA], endotoxin, diamine oxidase [DAO], and zonulin) by one-way analysis of variance (ANOVA) at G95d and L10d. At L10d, 4% FBF decreased the content of DAO, and endotoxin levels were significantly lower in serum samples from FBF-2 and FBF-3 (*P < *0.05, Fig. 2A and C), but there was no difference at G95d. Next, we examined three biomarkers—interleukin-6 (IL-6), tumor necrosis factor alpha (TNF-α), and IL-10—associated with inflammation. We found no differences at G95d. At L10d, the decreased serum IL-6 concentration was observed in sows fed 4% FBF compared to CON and FBF-1 (*P < *0.05, Fig. 2F). Sows in FBF-3 showed higher (*P < *0.05) serum concentrations of IL-10 than in CON and FBF-1, but not different from those in FBF-2. Serum TNF-α concentrations were lower (*P < *0.05) in FBF-3 than in CON. The effects of FBF on serum immunoglobulins in sows are presented in Fig. 2G and H. No differences were found in IgA and IgM contents, while serum IgG levels were considerably higher in FBF-2 and FBF-3 compared to CON (*P < *0.05, Fig. 2G). The same responses were also observed on L10d. In addition, we assessed inflammation, immunity, and growth-related biomarkers in piglet serum to see whether feeding sows with FBF-supplemented diets would influence the early development of piglets. We found no significant differences in IL-10 and TNF-α in piglet serum, while IL-6 was significantly lower in piglets of FBF-2 and FBF-3 groups (*P < *0.05, Fig. 3A). Dietary treatments did not alter serum concentrations of IgA in piglet serum, while the serum levels of IgG and IgM were tremendously improved in FBF groups (*P < *0.05, Fig. 3B). We found that the FBF-3 diet significantly reduced serum markers of systemic inflammation in sows and increased the level of serum immunoglobulins, as well as in piglets. Furthermore, the addition of FBF to the sow diet enormously increased the serum insulin-like growth factor concentration in piglets with FBF-2 (*P < *0.05) and FBF-3 (*P < *0.01). Meanwhile, there was no difference in growth hormone. These data indicate that the FBF diet reduced the levels of intestinal permeability biomarkers in sows, decreased inflammation levels, and increased immunoglobulin levels in sows and piglets. Based on these results, we found that FBF-3 had the most positive effect among all treatments.

### FBF-3 diet regulates short-chain fatty acids and gut microbiota composition in sows and piglets.

**FIG 4:**
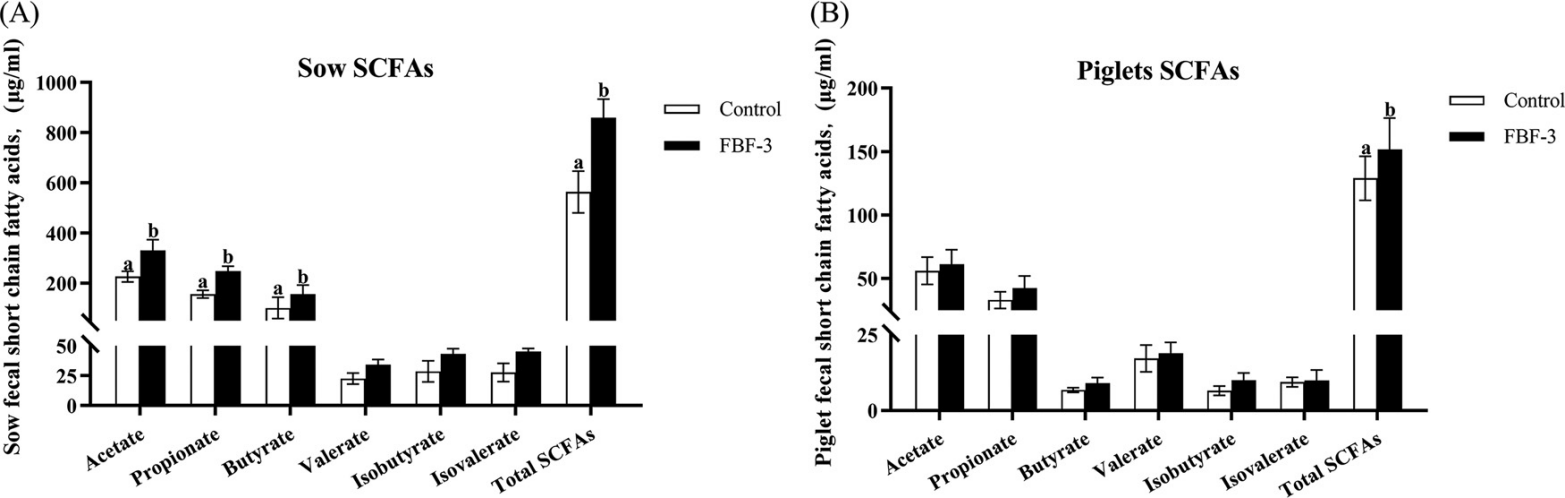
Effects of fermented bamboo fiber diet on fecal SCFAs in sows and piglets. (A) Sow fecal SCFAs on day 10 of lactation. (B) Piglet colonic contents contain SCFAs on day 21 of lactation. The data are presented as means ± the SEM (*n* = 6). Different letters mean significant differences (*P < *0.05). CON, wheat bran diet; FBF-3, 4% fermented bamboo fiber diet.

As described above, FBF-3 had the most positive effect among all treatments. Next, we measured the SCFA contents and microbiota composition in sow and piglet feces from the CON and FNF-3 groups. For sows, the contents of fecal acetate, propionate, butyrate, and total SCFAs were greatly improved in the FBF-3 group than those in CON (*P < *0.05, Fig. 4A). There were also no differences in the fecal concentrations of isobutyrate, valerate, and isovalerate among treatments. For piglets, there was no statistically significant except for total SCFAs (*P* > 0.05, Fig. 4B).

**FIG 5:**
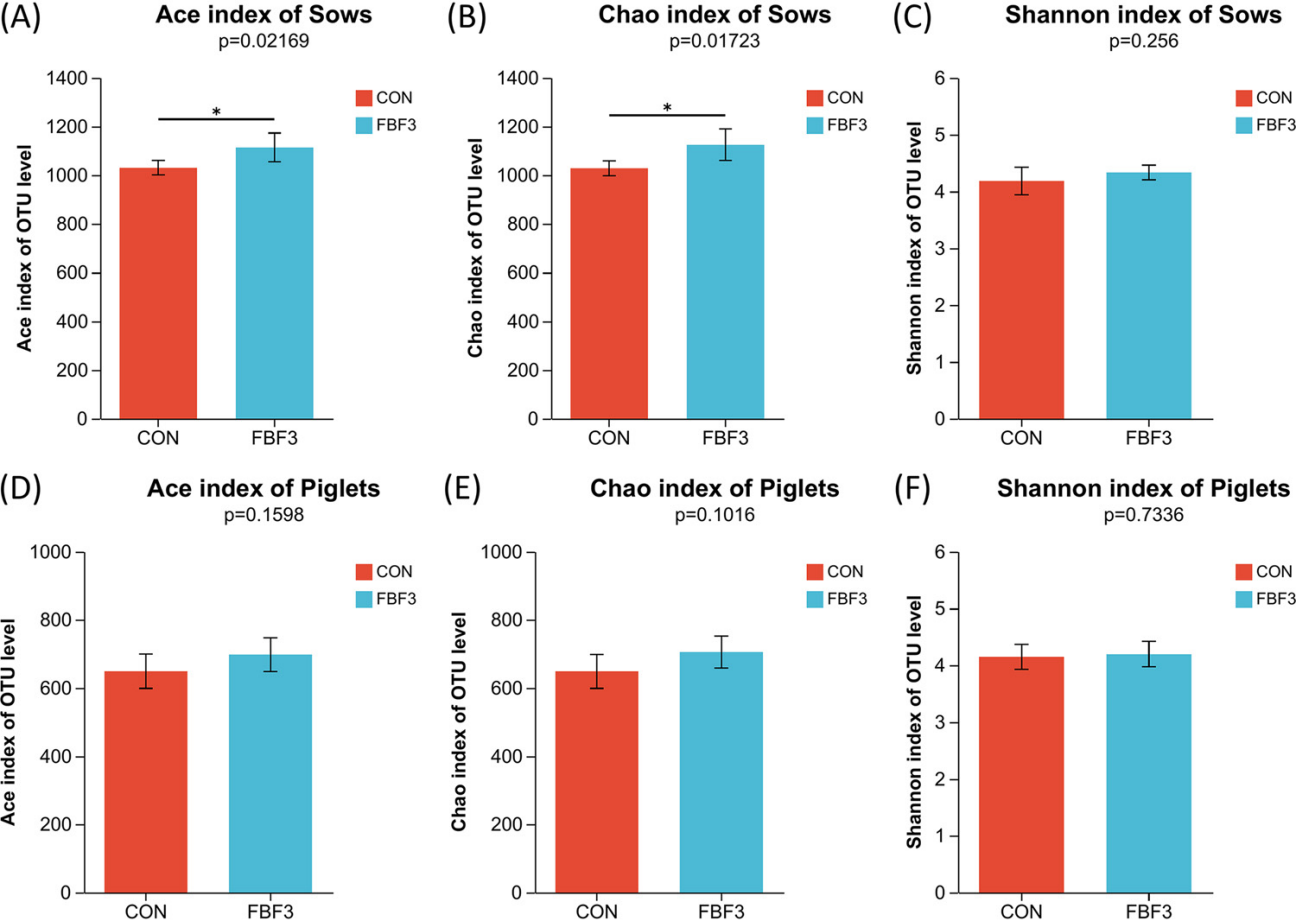
Description of gut microbial diversity in sow on day 10 of lactation and piglet at weaning. (A to C) Alpha-diversity is based on the Ace, Chao1, and Shannon indices of intestinal bacteria in sows. (D to F) Alpha-diversity is based on the Ace, Chao1 and Shannon indices of intestinal bacteria in piglets. Different letters mean significant differences (*, *P* < 0.05; *n* = 5). CON, wheat bran diet; FBF-3, 4% fermented bamboo fiber diet.

In light of the fundamental roles of the gut microbiota on host health. We analyzed the microbiota in sow feces at L10d and piglet colonic contents at L21d by deep sequencing of the V3-V4 region of the bacterial 16S rRNA gene. Compared to CON, the gut bacterial diversity was significantly increased in the FBF-3 sow group (*P < *0.05, Fig. 5A and B), as indicated by the Ace and Chao1 index values.

**FIG 6:**
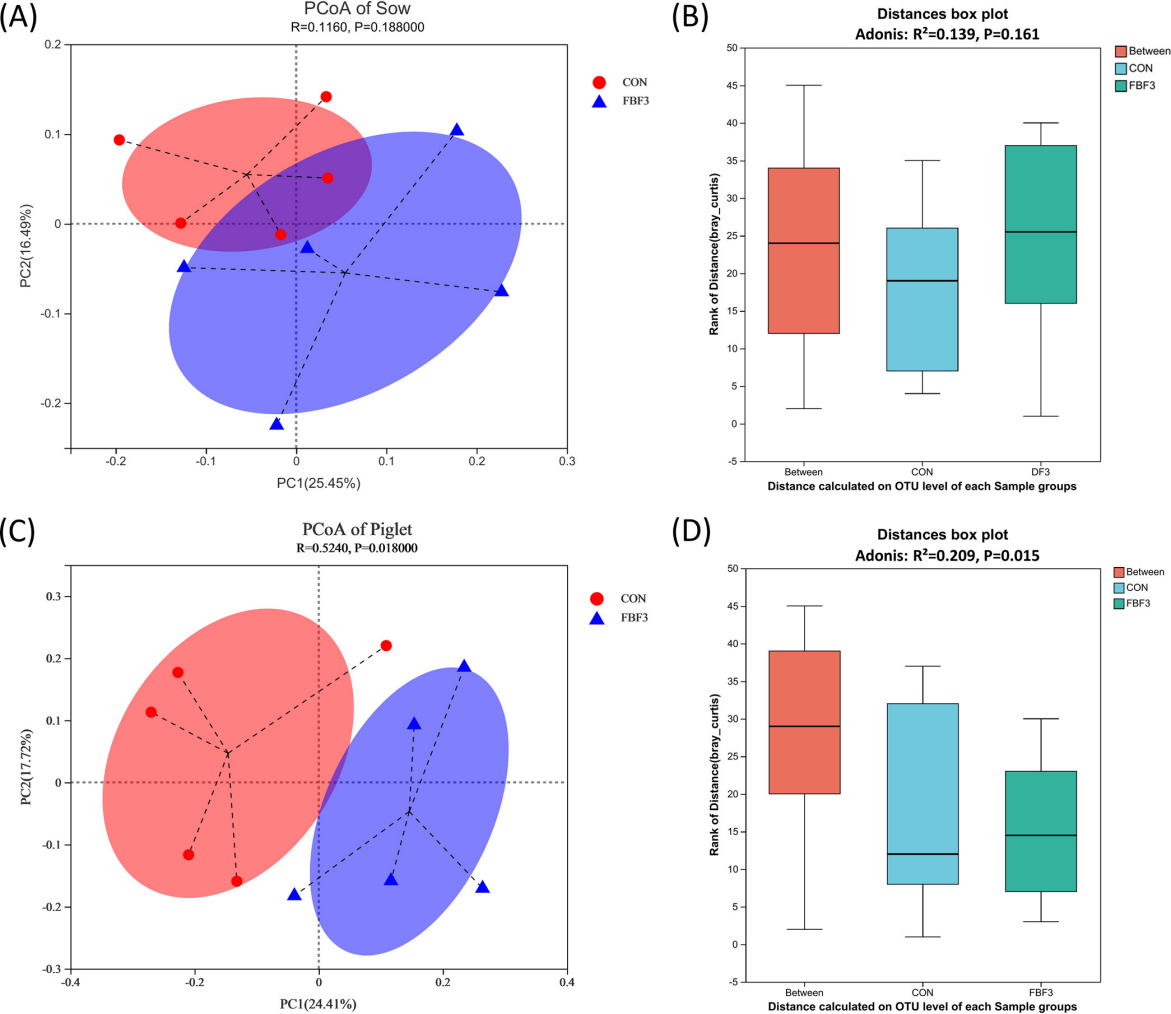
PCoA at the OTU level based on Bray-Curtis dissimilarity. (A) PCoA of sows on day 10 of lactation. (B) Distances box plot based on Adonis analysis on sows. (C) PCoA of piglets at weaning. (D) Distance box plot based on Adonis analysis on piglets. CON, wheat bran diet; FBF-3, 4% fermented bamboo fiber diet.

Principal coordinate analysis (PCoA) based on Bray-Curtis heterogeneity indicated that the microbial communities of piglets differed between treatment groups (Fig. 6C). In addition, the results of the analysis based on the Adonis test suggested that the differences in the beta-diversity of the intestinal flora of the piglets in the two groups were statistically significant by comparison (Fig. 6D).

**FIG 7:**
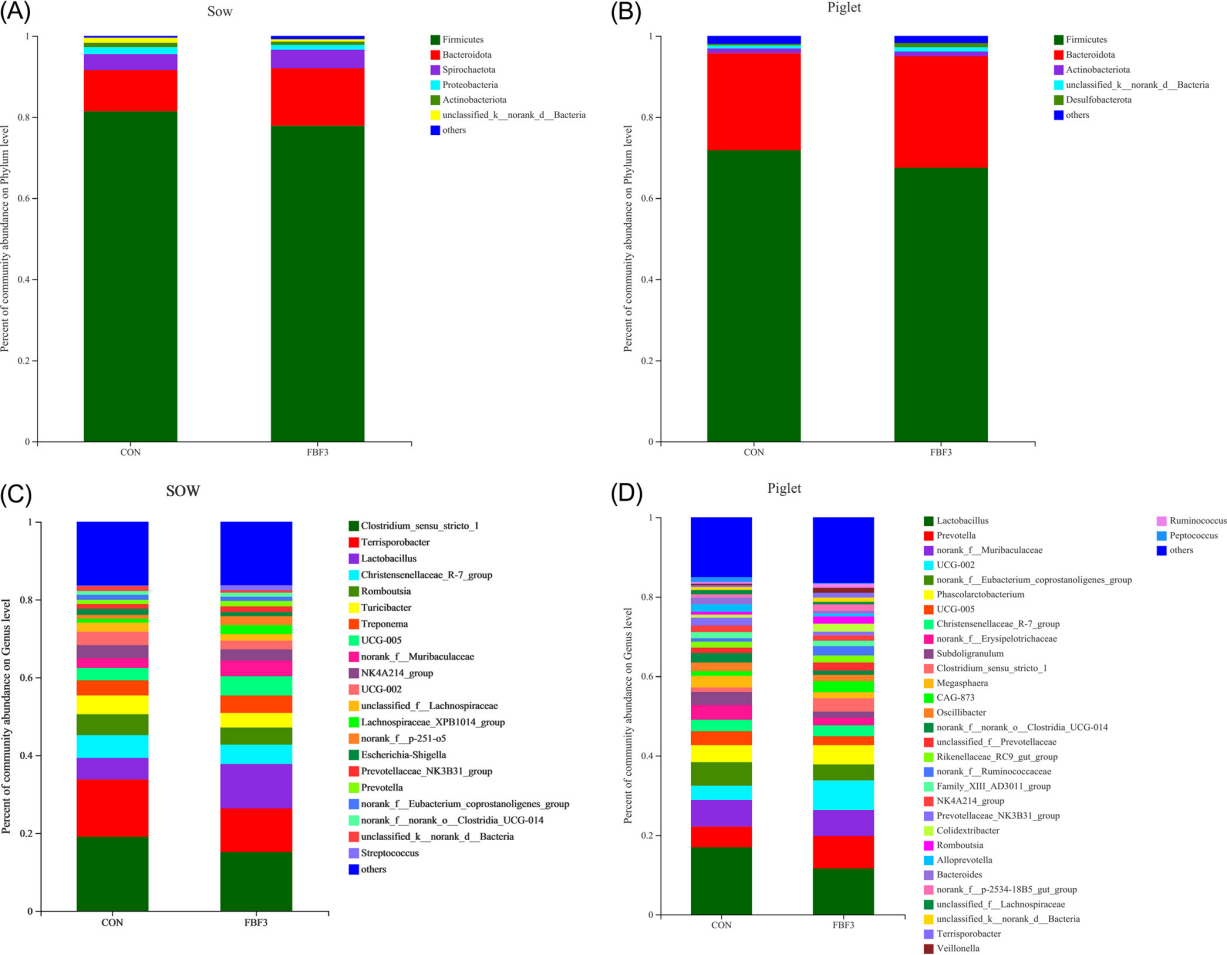
Description of gut microbial diversity in sows on day 10 of lactation and piglets at weaning. (A and B) Microbial community bar plot at the phylum level. (C and D) Microbial community bar plot at the genus level. CON, wheat bran diet; FBF-3, 4% fermented bamboo fiber diet.

Subsequently, the gut microbial composition of sows and piglets were studied. Taxonomic bar charts showed that the dominant phyla in sows were *Firmicutes*, *Bacteroidetes*, and *Spirochaetes*, accounting for more than 95% (Fig. 7A). *Firmicutes*, *Bacteroidetes*, and *Actinobacteriota* are the prevailing bacteria in piglet intestinal microbiota (Fig. 7B). On L10d, the top three genera in the feces of sows in the CON group were *Clostridium_sensu_stricto_1* (19.1%), *Terrisporobacter* (14.7%), and *Christensenellaceae_R-7_group* (5.9%); those in the FBF-3 group were *Clostridium_sensu_stricto_1* (15.1%), *Lactobacillus* (11.4%), and *Terrisporobacter* (11.2%) (Fig. 7C). For piglets, the dominant genera in CON were *Lactobacillus* (16.9%) *norank_f__Muribaculaceae* (6.7%), and *norank_f__Eubacterium_Coprostanoligenes_Group* (5.9%). Those in the FBF-3 group were *Lactobacillus* (11.6%), *Prevotella* (8.2%), and *UCG-002* (7.4%) (Fig. 7D).

**FIG 8:**
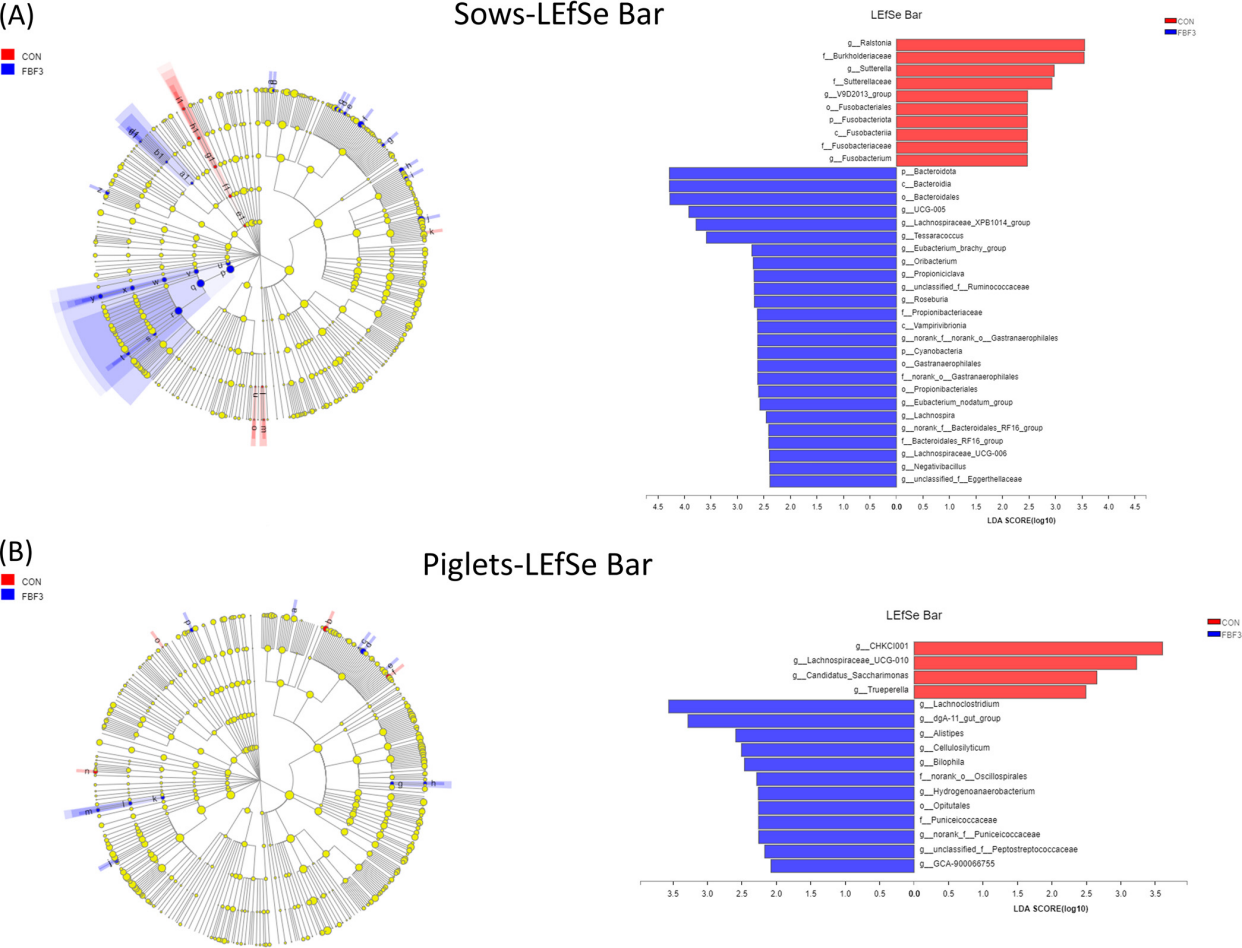
(A and B) LEfSe analysis for sows (A) and piglets (B) determined using a one-against-all (less strict) approach. Different color mean significant differences (*P* < 0.05; *n* = 5). CON, wheat bran diet; FBF-3, 4% fermented bamboo fiber diet.

Further linear discriminant effect size (LEfSe) analysis showed that, at L10d, an FBF diet led to a significant increase in the relative abundances of *UCG-005*, *Lachnospiraceae_XPB1014_group*, *Tessaracoccus*, *Eubacterium_brachy_group*, *Oribacterium*, *Propioniciclava*, *unclassified_f__Ruminococcaceae*, *Roseburia*, *norank_f__norank_o__Gastranaerophilales*, *Eubacterium_nodatum_group*, *Lachnospira*, *norank_f__Bacteroidales_RF16_group*, *Lachnospiraceae_UCG-006*, *Negativibacillus*, and *unclassified_f__Eggerthellaceae* and a reduction in the relative abundances of *Ralstonia*, *Sutterella*, *V9D2013_group*, and *Fusobacterium* in sows fed a 4% FBF supplemented diet compared to the CON group (Fig. 8A). Next, LEfSe analysis of piglets showed that *Lachnoclostridium dgA-11_gut_group Alistipes*, *Cellulosilyticum*, *Bilophila*, *Hydrogenoanaerobacterium*, *norank_f__Puniceicoccaceae*, *unclassified_f__Peptostreptococcaceae*, and *GCA-900066755* largely increased. In addition, *CHKCI001*, *Lachnospiraceae_UCG-010*, *Candidatus_Saccharimonas*, and *Trueperella* dramatically reduced compared to the CON group (Fig. 8B). These results suggest that the addition of FBF to sow feed during late gestation and lactation altered the microbial composition of sows and piglets.

### The composition of gut microbiota of sows is associated with intestinal permeability and inflammatory response and affects piglet growth and intestinal health.

**FIG 9:**
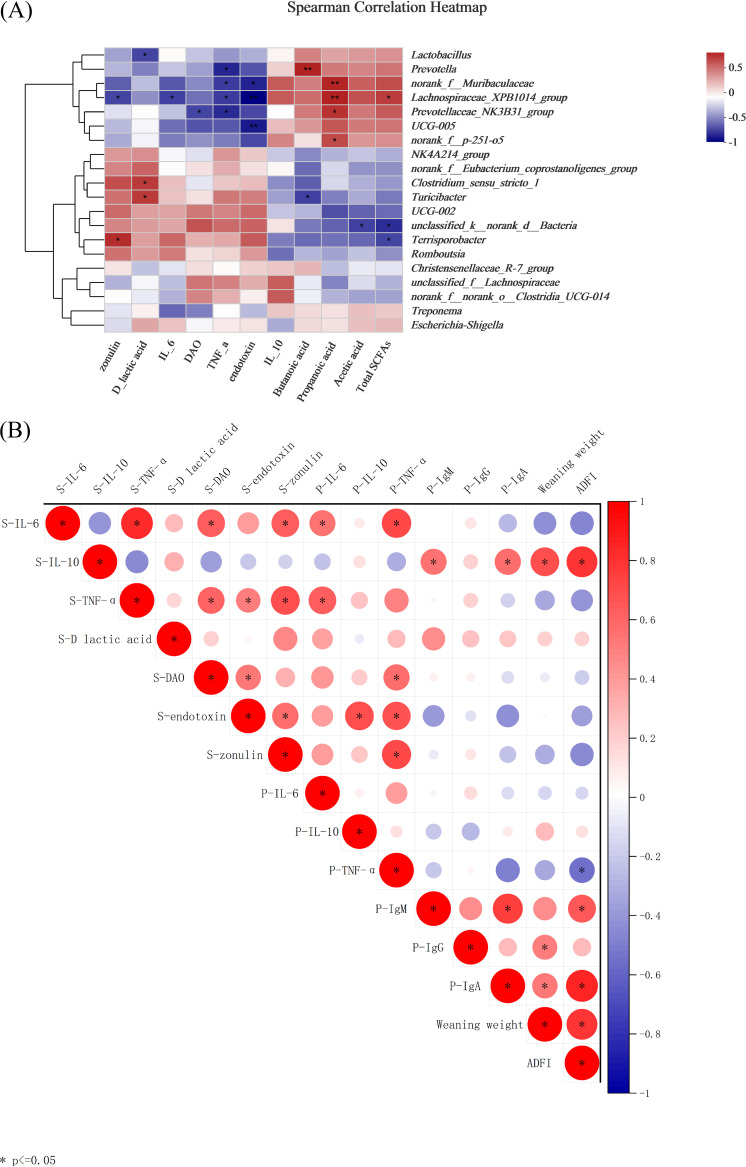
Association and model predictive analysis. (A) Spearman correlation analysis between host markers in sows. (B) Spearman correlation analysis between gut microbiota and host markers in sows. *, 0.01 < *P* ≤ 0.05; **, 0.001< *P* ≤ 0.01. Red indicates a positive correlation; blue indicates a negative correlation. S, sow serum; P, piglet serum.

As shown in Fig. 9A, the analysis of the correlation between microbiota based on LEfSe analysis and metabolic indicators at L10d showed that *Prevotella* had a positive correlation with fecal butanoic acid (*P < *0.01, *R *= 0.78) and a negative correlation with serum TNF-α (*P < *0.05, *R *= –0.73). *norank_f__Muribaculaceae* was positively correlated with propanoic acid (*P* < 0.01, *R* = 0.77) and negatively correlated with serum TNF-α (*P* < 0.05, *R* = –0.66) and endotoxin (*P < *0.05, *R* = –0.74). The anti-inflammatory bacteria of the *Lachnospiraceae_XPB1014_group* had a significant positive correlation with total SCFAs (*P* < 0.05, *R *= 0.68) and propanoic acid (*P < *0.01, *R *= 0.78) and was negatively correlated with serum endotoxin (*P < *0.01, *R *= –0.85), TNF-α (*P < *0.05, *R *= 0.66), IL-6 (*P < *0.05, *R* = –0.65), and zonulin (*P* < 0.05, *R *= –0.64). *Prevotellaceae_NK3B31_group* (*P < *0.01, *R *= 0.78) and *norank_f__p-251-o5* (*P < *0.05, *R *= 0.64) were both positively correlated with propanoic acid; conversely, *Prevotellaceae_NK3B31_group* was also negatively correlated with serum TNF-α (*P < *0.05, *R *= –0.72) and DAO (*P < *0.05, *R *= –0.64). *Lactobacillus* was negatively correlated with serum DLA (*P < *0.05, *R *= –0.64), but *Clostridium_sensu_stricto_1* (*P < *0.05, *R *= 0.70) and *Turicibacter* (*P < *0.05, *R *= 0.70) were positively correlated with it. Similarly, *Turicibacter* was negatively correlated with butanoic acid (*P < *0.05, *R *= –0.65). *unclassified_k__norank_d__Bacteria* was negatively correlated with total SCFAs (*P < *0.05, *R *= –0.71) and acetic acid (*P < *0.05, *R *= –0.65). In the same way, the inflammatory bacterium *Terrisporobacter* was also negatively correlated with total SCFAs (*P < *0.05, *R *= –0.64) and positively correlated with serum zonulin (*P < *0.05, *R *= 0.75).

Spearman correlation analysis showed that AFDI of lactating sows was significantly and positively correlated with serum IL-10 (*P < *0.05, *R *= 0.76), piglet weaning weight (*P < *0.05, *R *= 0.62), and piglet serum IgM (*P < *0.05, *R *= 0.45) and IgG (*P < *0.05, *R *= 0.46) and negatively correlated with piglet serum TNF-α (*P < *0.05, *R *= 0.62). Equally, inflammatory factors were associated with the intestinal barrier in sows, and serum TNF-α was significantly and positively correlated with IL-6 (*P < *0.05, *R *= 0.80), DAO (*P < *0.05, *R *= 0.47), endotoxin (*P < *0.05, *R *= 0.53), and zonulin (*P < *0.05, *R *= 0.62). In piglets, serum TNF-α showed positive correlations with sow serum IL-6 (*P < *0.05, *R *= 0.73), DAO (*P < *0.05, *R *= 0.55), endotoxin (*P < *0.05, *R *= 0.63), and zonulin (*P < *0.05, *R *= 0.73) and negative correlations with sow ADFI (*P < *0.05, *R *= –0.86) (Fig. 9B).

**FIG 10:**
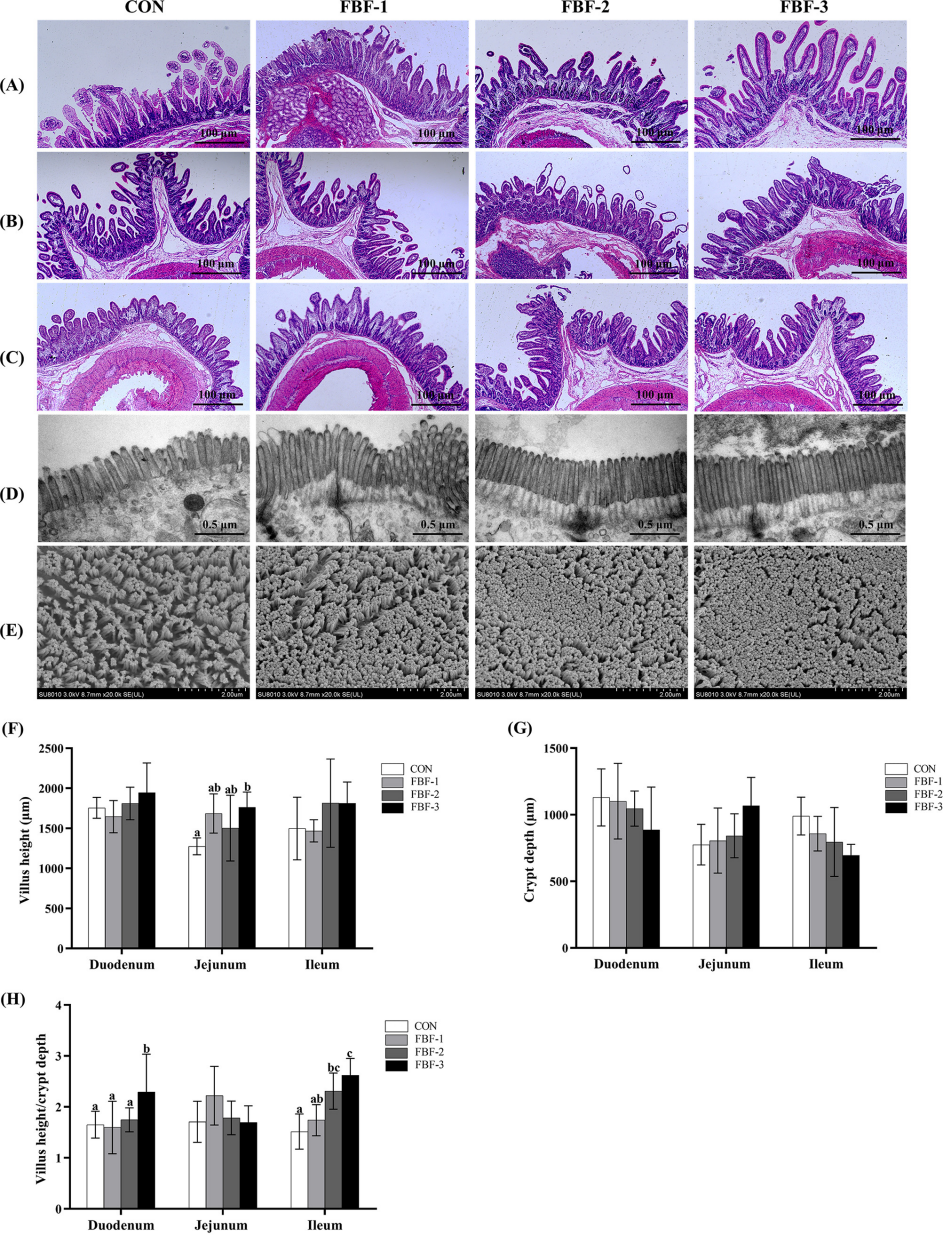
Histomorphological images of the intestinal tract of piglets. The duodenum (A), jejunum (B), and ileum (C) were stained with H&E. Scale bar, 100 μm. (D) TEM images of the jejunum structure. Scale bar, 0.5 μm. (E) Scanning electron micrograph images of the jejunum. Scale bar, 2 μm. (F) Villus height (VH); (G) crypt depth (CD); (H) villus height/crypt depth (V/C ratio).

The intestines of piglets are immature and incomplete, and their structures and functions are susceptible to damage from various stresses, infections, and food-related factors. To evaluate the effects of FBF on the development of the intestinal tract of the offspring, histomorphologic images of the piglet intestine were observed. As shown in Fig. 10F, the height of the jejunal villi was significantly increased in the FBF-3 group (*P < *0.05), whereas the crypt depth was no different among different groups. In particular, the villus height/crypt depth (V/C) of the duodenum and ileum in FBF-3 increased compared to the CON group (*P < *0.05, Fig. 10H). Furthermore, slightly broken microvilli were observed in the tissues obtained from the CON group (Fig. 10D and E). Based on the improved villus height and V/C, we concluded that a sow FBF diet may improve the intestinal morphology of piglets.

## DISCUSSION

Sows mobilize their body reserves to support milk synthesis during pregnancy and lactation, which generally leads to metabolism and immunity undergoing drastic changes. Maternal nutritional status plays a fundamental role in offspring gut health programming (30). Dietary fiber as a functional food can positively regulate gut health. Typically, different sources of dietary fiber have different effects depending on their physicochemical properties and fermentation type (10, 31–33). In the present study, a novel fermented bamboo fiber improved the performance of sows and their offspring. Namely, FBF modulated sow gut microbiota and immune status and alleviated inflammation, which was positively related to sow and piglet performance. Interestingly, our results showed the beneficial effects of FBF-3 are the best among experimental groups.

In the late stages of pregnancy, sows cease fat deposition and mobilize stored fat and energy, which is sent to the mammary gland for milk synthesis and generally results in body weight loss at weaning (34, 35). However, maintaining the backfat thickness and body condition of sows during late gestation and lactation is proven to be one of the most important factors determining subsequent reproductive performance (36). In addition to reducing body weight losses, the metabolism and immune changes in sows during pregnancy and lactation also affect the growth of piglets; increasing sow lactation feed intake maintains body condition and milk production, which directly improves reproductive performance (37, 38). Previous studies have shown that dietary fiber supplementation not only promotes satiety in sows but also improves sow feed intake during lactation (12, 34, 39). In the present study, we found that the addition of different levels of FBF to the diet of sows in late gestation and lactation affected the performance of both sows and piglets, with 4% FBF in particular significantly increasing ADFI in sows and weaning weight in piglets. Meanwhile, FBF notably reduced the backfat loss and constipation rate of sows. These results are consistent with those from other studies. Shang et al. observed a lower lactation weight loss in sows fed a higher-fiber diet compared to those fed the control diet (40). Liu et al. demonstrated that different sources of fiber improved sow and litter performance compared to CON (34). Sun et al. demonstrated that high-fiber diets containing konjac flour or alfalfa meal positively affect sow and litter performance (41). To summarize these findings, it has been demonstrated that including both soluble and insoluble fiber in sow prenatal meals can boost feed intake during lactation and affect litter performance, albeit via different mechanisms. Here, we found that the backfat loss of sows remarkably decreased in the FBF-3 groups comparison to the CON groups. The tendency for increased ADFI and decreased backfat loss in FBF groups in this study suggests that a suitable added amount of bamboo fiber may have been close to 4%. Body energy reserve and milk output have a positive relationship with the higher voluntary feed intake of lactating sows, which directly affects piglet growth performance (37). Compared to CON, FBF treatments remarkably increased the solid content of the colostrum. Moreover, 4% FBF showed significant improvement in the milk yield and protein contents of ordinary milk.

Constipation is a common symptom for pregnant sows because the relatively high volume of the fetus results in decreasing gastrointestinal motility and prolonging transit time, thereby resulting in increased water absorption and eventually low frequency and hard stools (10, 40). Also, constipated sows have a higher incidence of developing mastitis than those without constipation (42). FBF is rich in indigestible polysaccharides (82.38%), which can help treat constipation mainly by regulating gut microbiota composition, the synthesis of gut hormones, and water absorption, as well as SCFA production (25). To give an illustration, BSH-1, which is an O-acetylated xylan obtained from bamboo shavings, significantly shortened the defecation time and raised the gastrointestinal transit rate, stool production, and cecal concentration of SCFAs and effectively relieved L loperamide-induced constipation in mice (29). The positive effect of FBF on stool water content and constipation observed in this study may be related to the physicochemical properties (water holding capacity, fermentability, etc.) of dietary fiber. DF is rapidly fermented with the production of gases and increases the microbial biomass, which keeps high the water-holding capacity of stools (37). All fermentable DF is consumed as food moves toward the distal colon, and stool mass and water content with regard to reducing colon transit time are determined by insoluble, poorly fermented DF (31). It is generally believed that constipation can lead to complications such as fecal impaction, bowel perforation, and hemorrhoids, accompanied by inflammation in the gut mucosa (43, 44). Likewise, a previous study also indicates that mothers were found to have symptoms of metabolic syndrome during late gestation and lactation, mainly characterized by low-level inflammation and metabolic disorder (45). Moreover, the microinflammatory state decreases the diversity of intestinal flora and accelerates the ecological imbalance of intestinal flora (46, 47). Here, lower serum concentrations of gut permeability biomarkers (endotoxin and DAO) and proinflammatory cytokines (IL-6 and TNF-α) were observed in sows fed 4% FBF during lactation. Endotoxin is a lipopolysaccharide present at high concentrations in the gut, serum, and other tissues during bacterial infection (38). DAO protects the mucosal surface of the small intestine from cholera pathogens and reflects the integrity of the intestinal mechanical barrier and the degree of damage sustained (48). Serum TNF-α and IL-6 are indicators of proinflammatory reactions. Sows fed 4% FBF during lactation also had higher serum concentrations of anti-inflammatory cytokine IL-10 than did those fed Wheat Bran. It is known that IL-10, an anti-inflammatory cytokine, exerted anti-inflammatory function by decreasing cytokine and chemokine production (4). Therefore, it is suggested FBF in the sow diet was found to lower gut permeability and reduce gut and systemic inflammation. Anti-inflammatory effects of bamboo fiber have, to our knowledge, not yet been reported in pigs. However, these findings are in agreement with research in mice, where an O-acetylated xylan obtained from bamboo significantly reversed the expression of inflammation-related genes in constipated mice (29). In the present study, the increased concentration of IgG was also reflected in a better immunological response for sows. The IgG and IgM levels in the serum of piglets in the FBF group were largely increased which suggested that sow diet FBF had a beneficial effect on their offspring’s immunity. Piglets from FBF-3 and FBF-2 also have lower serum concentrations of IL-6. Furthermore, Spearman correlation analysis revealed that there was a significant positive correlation between IL-6 in piglets and sows. TNF-a expression in piglets has positively correlated with IL-6, zonulin, and endotoxin expression in sows and negatively correlated with ADFI during lactation. On the other hand, IL-10 expression in sows was positively correlated with piglet IgG levels, weaning body weight, and sow ADFI. It can be concluded that the performance improvements observed in sows fed with an FBF diet and their piglets are due to the alleviation of gut permeability and inflammatory reactions, as well as improved physical health. As a result, it is crucial to reduce inflammatory responses in sows during late pregnancy and lactation, as is ensuring that normal metabolic immunological changes occur over the sow reproductive cycle. The positive effect of FBF on inflammation and immunomodulatory capacity observed in this study may be related to the changes in intestinal microbiota and microbial metabolites.

It has been suggested that changes in the intestinal microbiota and its metabolites mediated by dietary fiber may play an important role in maintaining intestinal microecological homeostasis and protecting intestinal health (9). Certain bacterial species in the gut ferment indigestible dietary fibers into SCFAs, such as acetate, propionate, and butyrate, to mediate host energy balance, immune modulation, and mucosal barrier function (7, 49, 50). We do know that the gut microbial product butyrate upregulates the expression of tight junction proteins and induces the differentiation of Tregs in the colonic mucosa to maintain gut homeostasis (51). For instance, dietary fiber can prevent and treat intestinal inflammation induced by a high-carbohydrate and low-fiber western diet in mice by repairing the damaged intestinal mucus layer (29). The protection provided by fiber and resistant starch in experimental colitis is thought to be dependent on the production of SCFAs by intestinal microbes (9, 10). In the present study, FBF showed a greater positive effect than Wheat Bran, implying that FBF may provide benefits by modulating the gut microbiota and SCFAs, especially acetate and butyrate, in the FBF group. Butyrate could attenuate intestinal inflammation through upregulation of cytokine IL-10 expression by regulatory B cells (Bregs) according to *in vitro* data (52), and IL-10 showed a positive correlation with butyrate in this experiment, while the insoluble fiber can be slowly fermented in the hindgut and has beneficial effects throughout the intestine (53, 54). Acetate, propionate, butyrate, and total SCFA contents were greatly promoted in the FBF-3 group. This suggests greater microbial fermentation in the gut. PCoA showed significant clustering between the two dietary treatments, indicating that microbial composition varied depending on the source of fiber. In most mammals, the gut microbiota is typically dominated by *Firmicutes* and *Bacteroidetes* (55). Likewise, *Firmicutes* and *Bacteroidetes* were indeed the two major phyla found in the feces of sows and piglets. In general, the Chao1 value and Ace value can be used as indicators of species richness in a community (39). Increased microbial diversity contributes to improving the intestinal condition and preparing the physiology for the next cycle (56). Here, Chao1 and Ace indices indicated the addition of 4% FBF led to an increase in sow fecal bacterial abundance compared to the CON group. LEfSe analysis showed that the addition of FBF to the diets of sows in late gestation and lactation significantly reduced the relative abundance of proinflammatory bacteria (Fig. 5E). *Fusobacterium* is considered to be an inflammatory microorganism and a prognostic biomarker that inhibits T-cell responses and promotes the expression of inflammatory factors and has recently been implicated in colorectal cancer (57, 58). *Fusobacterium* remarkably decreased in FBF-3-treated groups (*P < *0.05, Fig. 5E). Other pathogens, such as *Sutterella*, are also downregulated in FBF-3-treated groups (*P < *0.05, Fig. 5E). The family *Sutterellaceae* has been associated with intestinal inflammation. *Sutterella* could degrade IgA and impair the functionality of the intestinal antibacterial immune response, and pass on susceptibility to intestinal diseases, such as inflammatory bowel disease, to offspring (59). As a result of intestinal permeability inhibition caused by endotoxin or exotoxin, these inflammatory genera can lead to bacterial translocation, endotoxemia, and low-grade inflammation (60). Another key thing to remember the pathway from polysaccharides to SCFAs is a complex and indirect network of metabolism. Some bacteria may be considered keystone species because they initiate the breakdown of specific substrates. It has been reported that *Bacteroidales* can degrade cellulose, and their genomes are effective in encoding the decomposition of high-molecular-weight plant polysaccharides (61, 62). For example, *Ruminococcaceae* can help degrade resistant starch-liberated, which are then metabolized by the microbial community (63). The present study demonstrated that sows fed FBF showed a greater abundance of *unclassified_f__Ruminococcaceae*. Furthermore, the family *Lachnospiraceae* (which includes the genus *Lachnospira*), *Roseburia*, *Oribacterium Lachnospiraceae_UCG-006*, and *Lachnospiraceae_XPB1014_group* are known to be SCFA-producing bacteria (64). *Lachnospira*, an acetate-producing bacteria, can regulate intestinal local pH to maintain the stability of the chemical barrier (51). The butyrate-producing genus *Roseburia* is administered to improve the gut ecosystem and prevent leaky gut (65). Butyrate-producing *Lachnospira* and *Roseburia* are potential markers of health (37). For the *Lachnospiraceae_XPB1014_group*, significant positive correlations were found in cellulosic degradation (66). *Oribacterium* ferments glucose and produces acetate as a metabolic by-product (20). Our results suggest that the addition of FBF to the diet of sows in late gestation and lactation can regulate the gut microbiota and the production of SCFAs, thus improving the gut health of sows. In addition, the correlation analysis further revealed relationships between sow gut microbiota, gut permeability markers, serum inflammatory factors, and SCFAs. The concentrations of acetate, propionate, and butyrate were positively correlated with anti-inflammatory bacteria, including *Lachnospiraceae_XPB1014_group*, *norank_f__Muribaculaceae*, and *Prevotella*, which were also associated with carbohydrate digestion and SCFA production (39, 40, 67). We also found bacteria that are related to metabolic indicators, such as *Lachnospiracea_XPB1014_group*, which is also significantly enriched in FBF-3. However, SCFA production was negatively correlated with the anti-inflammatory bacterium *Terrisporobacter*. Overall, supplementation with FBF altered the metabolism of nonstarch polysaccharides and the synthesis of SCFAs by the microbiota, which appeared to promote the cultivation of beneficial bacteria and reduce the relative abundance of proinflammatory bacteria.

Gut development in offspring is greatly affected by the maternal gut microbiota and breast milk (19). Studies have shown that the fiber content of the mother’s diet may have some influence on the microbiota of the offspring (15, 45). A mature gut microbiome in suckling offspring is beneficial to host growth and development and prepares animals for weaning (18). Here, the effect of maternal dietary FBF supplementation on the assembly of gut microbial levels in lactating offspring was investigated. According to LEfSe analysis, a significant reduction in the genus level of certain pathogenic bacteria *Trueperella* was observed in piglets of the FBF-3 group. Some potential beneficial bacteria, such as *Alistipes* and *Lachnoclostridium*, showed a significant increase at the genus level. As an opportunistic pathogen, *Trueperella* may cause various septic infections such as gastroenteritis and can be used as a microbial marker to predict early diarrhea (68). *Alistipes* may have protective effects against some diseases, including liver fibrosis, colitis, cancer immunotherapy, and cardiovascular disease (69). *Lachnoclostridium*, a butyric acid producer, protects against human colon cancer (70, 71). Intestinal health, especially the integrity of the intestinal barrier, plays a crucial role in maintaining an organism’s healthy state (38). Small intestine morphology, including VH (villous height [VH]), CD (crypt depth [CD]), and the VH/CD ratio, have commonly been used as indicators in piglets (72). The nutrient uptake of the intestine mainly relies on its epithelium and microvilli. The results of this study showed that maternal dietary FBF supplementation positively changed piglet small-intestinal architecture (increased VH and VH/CD ratio). In summary, dietary FBF supplementation has been found to promote intestinal morphology development and increase the abundance of gut beneficial bacteria in piglets.

Given the importance of constipation, intestinal barrier, and systemic inflammation in perinatal metabolic syndrome in sows, our results identify the potential role of FBF as a low-cost local fiber resource that can improve sow and piglet performance and alleviate intestinal and systemic inflammation. FBF supplementation significantly increased the relative numbers of anti-inflammatory and DF degradation-associated bacteria and decreased the relative abundance of proinflammatory bacteria. In addition, 4% FBF was initially explored to have the most beneficial effect. Follow-up studies should further identify the most effective factors in the FBF complex and the mechanisms by which they regulate pig performance and health.

## MATERIALS AND METHODS

### Ethical approval.

All the procedures were approved by the Institutional Animal Care and Use Committee at Zhejiang University (ZJU2013105002) (Hangzhou, China) and were tested on a commercial pig farm located in Jinhua, Zhejiang Province, China.

### Fermented bamboo fiber.

Moso bamboo (*Phyllostachys edulis*), with an age of 2 to 3 years, was obtained from Zhejiang Province, China. The general bamboo powder production process involved chopping, crushing, and sieving with 40 mesh. The substrate for fermentation consisted of bamboo fiber powder and 1% glucose to compose a basal substrate, which was supplemented with sterile water to achieve a final moisture content of 40%. Bacillus subtilis (1 × 10^7^ CFU g^−1^) and *Saccharomycetes* (1 × 10^6^ CFU g^−1^) were added to the mixed substrate and fermented anaerobically in fermentation bags for 96 h. Also, the numbers of B. subtilis and *Saccharomycetes* increased to 3.12 × 10^7^ and 8.64 × 10^6^, respectively. A pH of 4.62 or lower in the FBF was finally observed. The nutrition determination of the fermented bamboo fiber and wheat bran is shown in Table 1.

### Experimental design.

Based on similar expected dates of confinement and backfat thickness, 60 healthy multiparous sows (Yorkshire × Landrace; 3 to 6 parity) were randomly allocated to the control (CON), 1.25%, fermented bamboo fiber (FBF-1), 2.5%, fermented bamboo fiber (FBF-2), and 4%, fermented bamboo fiber (FBF-3) groups. Each treatment included 15 sows. The control group used wheat bran as the main source of fiber. In order to investigate the appropriate amounts of FBF, we designed three concentration gradients of 1.25, 2.5, and 4% FBF (as-fed basis) to replace all the wheat bran in the sow’s diet; the 4% FBF was the same as the neutral detergent fiber (NDF) content of wheat bran in the CON. The 1.25% FBF and 2.5% FBF contained 30 and 60% of the NDF contained in wheat bran in the CON, and all nutrients were similar between the groups except for neutral detergent fiber. The experimental diets had almost similar content of NDF. All pregnant sows were supplied with feed formulated to meet or exceed the nutrient requirements for sows as recommended by NRC (2012) (see Table S2). Wheat bran and fermented bamboo fiber samples were dried at 110°C for 24 h, cooled, ground, and subjected to conventional nutrient analysis using AOAC international guidelines. The details of the nutrient composition of wheat bran and fermented bamboo fiber are presented in Table S1. On day 107 of pregnancy, sows were moved to individual farrowing pens with crates, slatted floors, and heat pads for the piglets. At parturition, the numbers of stillborn and liveborn piglets in each litter were recorded. In the 12 h after farrowing, the litter size and individual piglet birth weights were measured. When possible, litter sizes were adjusted to 11 to 12 piglets by adding or removing piglets within each dietary group without changing the mean litter birth weight. Lactating sows all consumed the same diet as during gestation. Both sows and piglets had free access to water. The sow backfat thickness and constipation from parturition to weaning and reproductive performance, as well as the growth performance and diarrhea incidence of piglets, were recorded.

### Sample collection.

Individual backfat thickness at the last rib was recorded for sows on day 90 of gestation, within 24 h after farrowing, and at weaning. The bamboo fiber was estimated at 7 cm from the backbone in a straight line from the tip of the last rib (P2) using ultrasonography (PIGLOG105, A mode scanner; SFK Technology A/S, Helver, Denmark). Within 12 h of farrowing, the numbers and weights of live piglets per litter were recorded, as well as the numbers of live piglets, body weights at weaning, and diarrhea incidence. During lactation, sows were fed *ad libitum*, and the daily feed intake was recorded by calculating the daily supply of feed minus the surplus feed. Six sows were randomly selected from each treatment, and one piglet from each litter with an average body weight was selected to collect serum, feces, and intestinal samples. Approximately 40 mL of milk per group was collected randomly on the day of delivery and day 7 and placed at −20°C before analysis. On day 95 of gestation and day 10 of lactation, the fresh blood was placed in 5-mL sterile, evacuated blood collection tubes that were then left to stand for 30 min and subsequently centrifuged at 3,000 × *g* for 10 min. The serum was stored in a freezer at −80°C until further analysis. Sow fecal samples were collected at 95 days of gestation and at 10 days of lactation. On day 21 after delivery, the piglets were sacrificed using electric shock (120 V, 200 Hz) to collect the colonic content (middle portion) and serum samples. The serum samples (10 mL) were collected in heparinized tubes from the vena jugularis of each sow and piglet. Plasma samples were then obtained by centrifuging the serum samples at 3,000 × *g* at 4°C for 10 min and stored at −80°C until analysis. Fresh fecal samples were collected individually from the pigs using sterile 10-mL centrifuge tubes and then frozen in liquid nitrogen.

### Stool water content and Milk composition.

In brief, the stool samples were dried at 70°C for 24 h, The water content of fecal samples was calculated as (wet weight – dry weight)/wet weight. The protein, fat, sugar, solids, and nonfat solids were used as nutritional markers to assess the quality of milk with an automatic milk composition analyzer (Nanjing, China).

### Serum parameters.

Serum samples were thawed at 4°C and mixed thoroughly before analysis. Serum concentrations of immunoglobulins (including IgA, IgG, and IgM) and inflammatory cytokines (including IL-6, IL-10, and TNF-α) were measured in sow and piglet serum samples. Endotoxin, zonulin, d-LA, and DAO were measured in sow serum samples, and growth hormone and insulin-like growth factor were measured in piglet serum samples. All serum indexes were measured by enzyme-linked immunosorbent assay kits according to the manufacturer’s instructions (Shanghai Enzyme-Linked Biotechnology, Shanghai, China). All assays were performed according to the manufacturer’s instructions.

### DNA extraction and 16S RNA sequencing.

Microbial DNA was extracted from fecal samples using the QIAamp DNA Stool minikit (Qiagen, CA, Hamburg, Germany) according to the manufacturer’s instructions. The purity and concentration of extracted DNA were quantified by using a NanoDrop 2000 spectrophotometer (Thermo Fisher Scientific, Waltham, MA). The V3-V4 hypervariable region of the 16S rRNA gene was amplified with barcoded primers 341F (5′-CCTAYGGGRBGCASCAG-3′) and R806 (5′-GGACTACNNGGGTATCTAAT-3′). Then, amplicons were extracted from 2% agarose gels and purified with a MinElute gel extraction kit (Qiagen, Dusseldorf, Germany) and assessed by using a QuantiFluor TM-ST fluorometer (Promega, Madison, WI). The construction of sequencing libraries was conducted by using a TruSeq DNA sample prep kit (Illumina, Madison, WI). Clean sequences were assigned to the same operational classification unit (OTU) using ≥ 97% similarity. Data analysis was performed on the free online platform of the Majorbio Cloud platform. Based on the OTU clustering analysis results, a variety of diversity indices can be performed. The alpha-diversity, including Shannon, Simpson, Sobs, Ace, Chao1, and coverage values, was calculated to reflect bacterial diversity and richness. The beta-diversity on unweighted UniFrac was calculated based on OTU levels. UniFrac-based principal-component analysis and PCoA were used to obtain and visualize principal coordinates from complex data. Community structure variability among samples was calculated by PCoA based on Bray-Curtis dissimilarity. The relative abundance of microbiota was examined at different taxonomic levels. The relative abundance of significant differences in phylum, class, order, family, and genus levels was calculated by LEfSe analysis.

### Fecal short-chain fatty acids.

Quantification of acetate, propionate, butyrate, isobutyric acid, valeric acid, and isovaleric acid in the fecal samples was performed using the following procedures. Briefly, ~0.5-g fecal samples were diluted in 8 mL of ultrapure water, homogenized by ultrasonic oscillation, and centrifuged at 12,000 × *g* for 10 min. The supernatant was then diluted 50 times and filtered through a 0.22-mm-pore-size filter, and 1.5 mL of supernatant was analyzed by using a gas chromatograph (7890A; Agilent, Carpinteria, CA) to determine the acetate, propionate, butyrate, isobutyrate, valerate, and isovalerate contents.

### Sample processing procedures of HE, SEM, and TEM.

4% paraformaldehyde was used to fix the fresh small intestine segments. The samples were processed by embedding, sectioning, and hematoxylin-eosin staining (H&E). Then, intestine morphology pictures were captured, and data (V, C, and V/C ratio) were measured using a microscope (Eclipse 80i; Nikon, Tokyo, Japan). Fresh animal intestinal segments were quickly stored in 2.5% glutaraldehyde fixative, followed by fixation with a 1% OsO_4_ solution. The samples were dehydrated with six concentration gradient concentrations of ethanol solution and dried in a Hitachi HCP-2 type critical point desiccator. The treated samples were examined using a Hitachi SU-8010 type scanning electron microscope. We used transmission electron microscopy to examine the samples that had undergone the same fixation and dehydration treatment. The samples were then permeabilized and embedded with a mixture of Spurr embedding agent and acetone and sliced through Leica EM UC7 ultrathin sectioning machine to obtain 70- to 90-nm sections for observation with a Hitachi H-7650 transmission electron microscope.

### Statistical analysis.

Statistical analyses were performed using SPSS 20.0 software (IBM, New York, NY). Data were evaluated by one-way ANOVA, and the differences between means were assessed using Duncan’s test. A *P* value of 0.05 was considered statistically significant. Spearman correlation analysis was performed with OriginLab 2022 software (OriginLab Corp., Northampton, MA). The data were evaluated by Spearman correlation analysis of the Euclidean distance. The sample size and sample individuals in the present study for Spearman correlation analysis corresponded one-to-one between productive performance inflammatory response parameters and intestinal barrier parameters (*n* = 5). The results of the Spearman correlation analysis are shown in the heat map (*, *P < *0.05; **, *P < *0.01).
