# Supplementary material for: Fermented Bamboo Fiber Improves Productive Performance by Regulating Gut Microbiota and Inhibiting Chronic Inflammation of Sows and Piglets during Late Gestation and Lactation
Source: Microbiol Spectr. 2023 Apr 12;11(3):e04084-22. doi: 10.1128/spectrum.04084-22 (PMC10269633; doi:10.1128/spectrum.04084-22)
Supplement: Supplemental file 1 — Tables S1 to S3. Download spectrum.04084-22-s0001.pdf, PDF file, 0.1 MB [file spectrum.04084-22-s0001.pdf]

TABLE S1 Nutrient composition of Fermented bamboo fiber and Wheat bran (%, as-fed basis)

Item	Fermented bamboo fiber	Wheat bran
DM, %	78.32	87.0
CP, %	1.35	13.66
EE, %	0.30	3.39
Ash, %	0.97	4.26
NDF, %	64.52	32.19
ADF, %	48.15	11.31
CF, %	64.25	5.66
Lignin, %	12.11	5.64
Ca, %	0.34	0.10
Total P, %	0.24	0.80

DM, dry matter; CP, crude protein; EE, ether extract; NDF, neutral detergent fiber; ADF, acid detergent fiber; CF, crude fiber.

TABLE S2 Ingredient composition and nutrient concentration in the experimental (as-fed basis)

	Diet			
Items	Control	FBF-1	FBF-2	FBF-3
Ingredients, %				
Corn	60	65.75	63.5	61.08
Soybean meal, dehulled	20	24	22	21
Wheat bran	8	–	–	–
Fermented bamboo fiber	–	1.25	2.50	3.92
Extruded soybean	8	5	8	10
98.5%L-Lys.Hcl	0.25	0.25	0.235	0.22
DL-Met	0.05	0.05	0.045	0.05
L-Thr	0.05	0.035	0.035	0.035
Limestone	1.2	1.2	1.2	1.2
$Ca(HCO_3)_2$	1.4	1.4	1.4	1.4
NaCl	0.40	0.40	0.40	0.40
Premix	1.0	1.0	1.0	1.0
Total	100	100	100	100
Nutrition composition				
DE (MJ/Kg)	13.36	13.42	13.40	13.36
CP (%)	17.89	17.83	17.82	17.89
NDF (%)	12.48	11.5	12.30	13.35
Lys (%)	1.05	1.05	1.05	1.05
Met+Cys (%)	0.64	0.64	0.64	0.64
Threonine (%)	0.75	0.76	0.75	0.75
Ca (%)	0.85	0.85	0.84	0.84
AP (%)	0.39	0.39	0.38	0.38

a. Analysed values determined in duplicate.

b. Premix provided per kilogram of diet: Gestation: vitamin A, 11,000 IU; vitamin D3, 1500 IU; vitamin E, 15 IU; vitamin K3, 1.6 mg; vitamin B1, 1.6 mg; vitamin B2, 3.1 mg; vitamin B6, 1.5 mg; vitamin B12, 0.04mg; niacin, 22.5 mg; D-pantothenic acid, 15 mg; folic acid, 2.5 mg; biotic, 0.2 mg; Fe, 80 mg; Cu, 7.5 mg; Zn, 95 mg; Mn, 35 mg; I, 0.5 mg; Se, 0.2 mg;

c. Thr, Threonine; CP, crude protein; EE, ether extract; NDF, neutral detergent fiber; ADF, acid detergent fiber; CF, crude fiber. Lys, Lysine; Met, Methionine; Cys, Cysteine; AP, Available phosphorus.

TABLE S3 Supplementary table for basic data of sows and piglets

Number	ADFI, (kg/d)	Average Wt at birth, (kg)	Average Wt at weaning, (kg)	Diarrhea incidence, (%)
0-1	6.42	1.11	5.21	1.12
0-2	6.46	1.18	5.57	1.34
0-3	6.40	1.4	5.3	1.32
0-4	6.19	1.19	5.6	1.32
0-5	6.33	1.24	5.32	1.44
0-6	6.41	1.22	5.52	1.4
1-1	6.63	1.52	6.2	1.17
1-2	7.00	1.08	5.4	1.19
1-3	7.34	1.34	5.18	1.08
1-4	6.50	1.31	5.51	1.09
1-5	6.78	1.25	5.5	1.11
1-6	6.96	1.29	5.64	1.1
2-1	7.25	1.06	5.41	1.03
2-2	6.98	1.33	5.84	1.17
2-3	6.78	1.27	5.5	1.03
2-4	7.87	1.19	6.37	1.07
2-5	7.12	1.17	5.73	1.08
2-6	7.32	1.21	5.83	1.05
3-1	7.00	1.26	6.12	1.06
3-2	7.46	1.08	6.09	0.98
3-3	7.86	1.35	6.12	1.03
3-4	7.91	1.45	6.2	1.04
3-5	7.51	1.06	6.04	1.02
3-6	7.61	1.38	6.22	0.99

CON: 0-1~0-6; FBF-1: 1-1~1-6; FBF-2: 2-1~2-6; FBF-3: 3-1~3-6.
